# Effects of Beta-Alanine Supplementation on Physical Performance in Aerobic–Anaerobic Transition Zones: A Systematic Review and Meta-Analysis

**DOI:** 10.3390/nu12092490

**Published:** 2020-08-19

**Authors:** Álvaro Huerta Ojeda, Camila Tapia Cerda, María Fernanda Poblete Salvatierra, Guillermo Barahona-Fuentes, Carlos Jorquera Aguilera

**Affiliations:** 1 Grupo de Investigación en Salud, Actividad Física y Deporte ISAFYD, Escuela de Educación Física, Universidad de Las Américas, sede Viña del Mar 2531098, Chile; danielbarahonaf@gmail.com; 2 Facultad de Ciencias, Escuela de Nutrición y Dietética, Magíster en Nutrición para la Actividad Física y Deporte, Universidad Mayor, Santiago 8580745, Chile; camitapiac@hotmail.com (C.T.C.); mariafernandapoblete@gmail.com (M.F.P.S.); 3 Facultad de Ciencias, Escuela de Nutrición y Dietética, Universidad Mayor, Santiago 8580745, Chile; carlos.jorquera@mayor.cl

**Keywords:** beta-alanine, ergogenic aid, physical performance, aerobic–anaerobic transition zone

## Abstract

Beta-alanine supplementation (BA) has a positive impact on physical performance. However, evidence showing a benefit of this amino acid in aerobic–anaerobic transition zones is scarce and the results controversial. The aim of this systematic review and meta-analysis is to analyze the effects of BA supplementation on physical performance in aerobic–anaerobic transition zones. At the same time, the effect of different dosages and durations of BA supplementation were identified. The search was designed in accordance with the PRISMA^®^ guidelines for systematic reviews and meta-analyses and performed in Web of Science (WOS), Scopus, SPORTDiscus, PubMed, and MEDLINE between 2010 and 2020. The methodological quality and risk of bias were evaluated with the Cochrane Collaboration tool. The main variables were the Time Trial Test (TTT) and Time to Exhaustion (TTE) tests, the latter separated into the Limited Time Test (LTT) and Limited Distance Test (LDT). The analysis was carried out with a pooled standardized mean difference (SMD) through Hedges’ g test (95% CI). Nineteen studies were included in the systematic review and meta-analysis, revealing a small effect for time in the TTT (SMD, −0.36; 95% CI, −0.87–0.16; I^2^ = 59%; *p* = 0.010), a small effect for LTT (SMD, 0.25; 95% CI, −0.01–0.51; I^2^ = 0%; *p* = 0.53), and a large effect for LDT (SMD, 4.27; 95% CI, −0.25–8.79; I^2^ = 94%; *p* = 0.00001). BA supplementation showed small effects on physical performance in aerobic–anaerobic transition zones. Evidence on acute supplementation is scarce (one study); therefore, exploration of acute supplementation with different dosages and formats on physical performance in aerobic–anaerobic transition zones is needed.

## 1. Introduction

A proper diet is one of the main factors in the improvement of physical performance. However, sometimes it is not enough to meet the energetic demands of training sessions [1]. For this reason and with the aim of maximizing physical performance, the use of nutritional supplements is widespread in sport [2], even more in younger athletes [3]. Nutritional supplements, such as protein and carbohydrates, are concentrated nutrient sources that substitute or complement the use of certain foods, while ergogenic aids, such as caffeine, creatine, or beta-alanine (BA), are pharmacological agents used with the aim of enhancing physical performance [4]. In this regard, one study showed that 48% of athletes use nutritional supplements and ergogenic aids [3], claiming that certain components, such as creatine, caffeine, sodium bicarbonate, and BA, contribute to an improvement in their physical performance [5,6,7].

Specifically, BA is a non-essential amino acid synthesized in the liver and found in products of animal origin [8]. Evidence shows that poultry, beef, and fish are products with a large BA content [9]. BA has been consistently shown to increase levels of carnosine (CA) in human skeletal muscle [9,10,11,12]. This last substance is synthesized by CA synthase when bonding BA with L-histidine [13]; CA is found in the muscular tissue and acts as a buffer of hydrogen protons (H^+^) in high-intensity physical exercises of short duration [11,14]. This is why athletes who follow a vegetarian diet will have lower muscular CA concentrations than those who follow an omnivorous diet [15].

When performing high-intensity exercises, due to the predominant energetic system (anaerobic metabolism of carbohydrates), a high release of H^+^ takes place, which leads to a decrease in pH [12]. This pH decrease can negatively affect the metabolic processes of phosphocreatine resynthesis, inhibit contractile processes, and diminish the glycolytic rate—all these factors contribute to the onset of muscular fatigue [14]. Some studies have concluded that an elevated muscular CA concentration could buffer between 8–15% of H^+^, opening the possibility of maximizing physical effort for a longer period of time [1]. On the other hand, other studies have shown that CA and L-histidine supplementation do not increase the bioavailability of intramuscular CA [5,14]. For this reason, and considering BA as a precursor in CA formation, several studies have shown an increase between 40–80% of intramuscular CA post BA supplementation [1,9,10,11,16]. In this regard, the acute effect of BA supplementation has been tested in doses of 30 mg·kg^−1^ of body mass and prolonged supplementation with doses ranging from 2.0 to 6.4 g/day for periods of time between 4 and 10 weeks [12,17]. At the same time, BA can be found as the main ingredient in multi-ingredient pre-workout supplements, although it is worth mentioning that these products have a lower dosage than that studied clinically [18]. Specifically, lower pH values have been measured after 4 min of high-intensity exercise [19] and the drop of pH is one of the factors responsible for the increase in ventilatory responses [20]. In parallel, the background shows that BA supplementation reported only one secondary effect, paresthesia [21,22]; this is a sensation of flushing associated with an irritant tingling in the ears, scalp, hands, and torso [23].

Related to pH stabilization, there are several studies that have used ergogenic aids to improve physical performance in aerobic–anaerobic transition zones [8,24]. The aerobic–anaerobic transition zone corresponds to an intensity range between aerobic threshold and anaerobic threshold [25] and may serve as a basis for assessing endurance performance individually as well as for prescribing intensities in endurance training [26]. In this regard, BA is among the ergogenic aids used to increase performance in aerobic–anaerobic transition zones [8,9,27]. One study has evaluated the effect of BA supplementation on physical performance, showing an improvement of 13.9% in ventilatory threshold [20]. In addition, another study reported that BA supplementation for 28 days enhanced sub-maximal endurance performance by delaying the onset of blood lactate accumulation (OBLA) [8]. However, other investigations have not found significant results in athletic performance [12], specifically in rowers [28], and trained cyclists [29] with BA supplementation.

The existing evidence shows controversial results that make it impossible to categorize or ensure that BA supplementation improves physical performance in aerobic–anaerobic transition zones (performance mainly connected to ventilator parameters). Hence, the primary aim of this systematic review and meta-analysis was to analyze the effects of BA supplementation on physical performance in aerobic–anaerobic transition zones. Likewise, the effects of different doses and supplementation times with BA were identified.

## 2. Materials and Methods

### 2.1. Literature Search Strategies

In order to perform this review, a thorough electronic search was carried out in several databases and search engines. Articles published in Web of Science (WOS), Scopus, SPORTDiscus, PubMed, and MEDLINE were included. A search limit was established from January 2010 to February 2020.

The bibliographic search was performed in accordance with the PRISMA^®^ statement guidelines for systematic reviews and meta-analyses [30]. In each of the aforementioned databases, the search included hits in the title, abstract, and key words search fields. The following key words were combined with Boolean operators AND/OR: [(“b-alanine” OR “beta-alanine” OR “b-alanine supplementation” OR “beta-alanine supplementation”) AND (“maximal aerobic speed” OR “maximal oxygen uptake” OR “maximal aerobic consumption” OR “endurance”)]. One of the authors performed the search, and two reviewed the studies. Together, they decided whether the studies were appropriate for inclusion.

### 2.2. Inclusion and Exclusion Criteria

The importance of each study was assessed according to the following inclusion criteria: (1) BA supplementation, either acute or chronic supplementation, (2) experimental design studies, (3) healthy adults, (4) studies that included physical performance evaluation in the aerobic–anaerobic transition zone (60–100% VO_2_max), (5) studies that included Time Trial Tests (TTT), or Time to Exhaustion (TTE) tests for physical performance evaluation, (6) studies that stated a baseline and control group, (7) studies showing negative and positive changes in TTT or TTE tests, and (8) studies published in English and Spanish. The studies that failed to fulfill the inclusion criteria were not considered in the systematic review nor the meta-analysis. Possible discrepancies were resolved through discussion until a consensus was reached.

### 2.3. Chronic and Acute Supplementation

Regarding the classification of the supplementation protocols assessed in this systematic review, acute supplementation was considered to be the one in which a unique dose of BA was used between 0 min and 24 h prior to physical exercise, while chronic supplementation was considered those protocols that used repeated dosages of BA for more than one day and up to 10 weeks [31]. 

### 2.4. Outcome Measures

The articles were examined regarding the effect of BA supplementation on physical performance in aerobic–anaerobic transition zones (60–100% VO_2_max) [26,32]. The primary outcome used for the systematic review and meta-analysis were (a) TTT and (b) TTE tests (Limited Time Test (LTT) and Limited Distance Test (LDT)). In order to establish the upper limit in the aerobic–anaerobic transition zone (100% VO_2_max), the minimum time used on the TTT and TTE test (LTT) was 300 s (the literature sets this as the minimum amount of time needed to determine VO_2_max) [33,34], while the minimum lower limit in the aerobic–anaerobic transition zone was 60% of VO_2_max [32]. These limits were set in order to include studies showing results of 5–63 min [21,35]. The systematic review and meta-analysis also included secondary outcomes stated in the studies. These secondary variables were (a) capillary lactate (mmol·L^−1^), (b) absolute VO_2_max (LO_2_·min^−1^), (c) HR (bpm), and (d) ratings of perceived exertion (RPE) according to the Borg scale [36]. It is important to mention that studies were excluded from the systematic review and meta-analysis if they only showed secondary results in the in extenso reading. Median values, standard deviations (SD), and sample sizes were included for the statistical analysis of the meta-analysis, for both the primary and secondary outcomes. If the selected studies did not include numerical data, it was requested of the authors, or if the data were plotted as figures, the values were estimated based on the pixel count. Additionally, the studies that declared paresthesia symptoms in their subjects were also included.

### 2.5. Publication Bias

**Figure 1:**
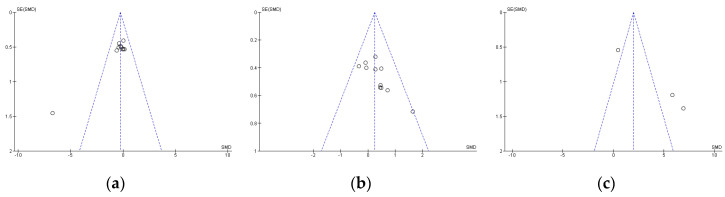
Standard error for Times Trial Test (**a**), Limited Time Test (**b**), and Limited Distance Test (**c**). SE: standard error; SMD: standardized median difference.

Publication bias was assessed using Egger’s statistical test. This test determined the presence of bias at *p* ≤ 0.05 [37]. Funnel plots were created to interpret the general effect, followed by an Egger’s statistic to confirm or refute publication bias. Egger’s analysis suggested that the primary variables did not show publication bias: (a) TTT: z = 1.35, *p* = 0.18; (b) LTT: z = 1.90, *p* = 0.06; (c) LDT: z = 1.85, *p* = 0.06 (Figure 1).

### 2.6. Quality Assessment of the Experiments

**Figure 2:**
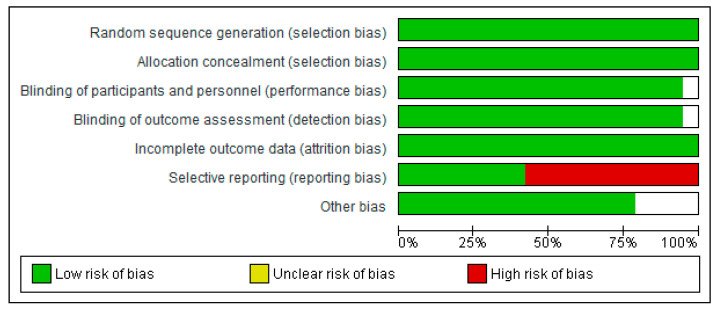
Risk of bias graph: review authors’ judgements about each risk of bias item presented as percentages across all included studies.

**Figure 3:**
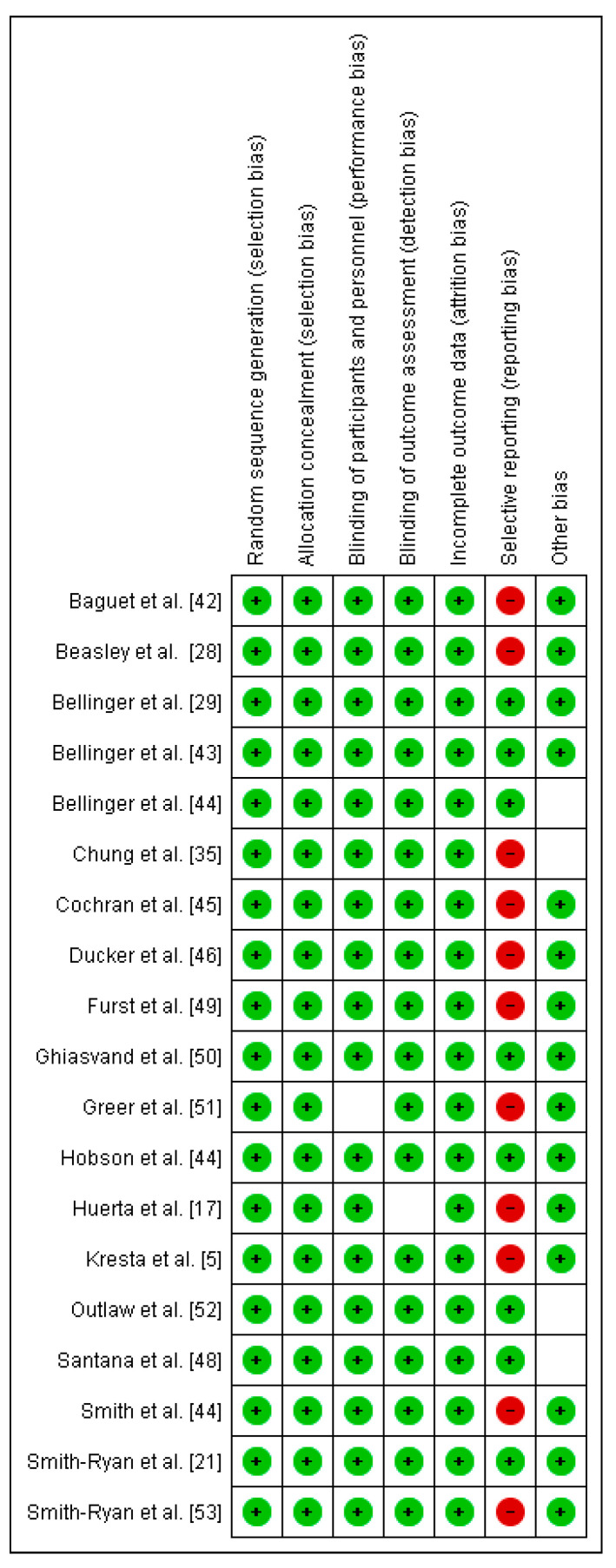
Risk of bias summary: review authors’ judgements about each risk of bias item for each included study.

The methodological quality and risk of bias for each selected study were assessed through a Cochrane Collaboration guideline [38]. The list was divided into six different domains: selection bias (random sequence generation, allocation concealment), performance bias (blinding of participants and personnel), detection bias (blinding of outcome assessment), attrition bias (incomplete outcome data), reporting bias (selective reporting), and other types of bias (declaration of conflict of interest). For each item, the answer to a question was considered; when the question was answered with a “Yes”, the bias was low; when it was “No”, the bias was high; when it was “Unclear”, the possible bias was connected to a lack of information or uncertainty. The full details of each study and domains are presented in Figure 2 and Figure 3.

### 2.7. Statistical Analysis

In order to evaluate the quality of the experiments and interpret the risk of bias values, Review Manager version 5.4 was used (Copenhagen: The Nordic Cochrane Centre, The Cochrane Collaboration, 2014). The same software was used to perform a descriptive and statistical analysis of the meta-analysis. To compare the supplementation of BA versus the placebo (PL), the number of participants, standardized mean difference (SMD), and standard error of SMD were analyzed for each study. Hedges’ g test was used to calculate the SMD of each study [39]. The overall effect and its 95% confidence interval (CI) were calculated by weighting the SMD by the inverse of the variance. Additionally, the SMD of both the BA supplemented and PL groups were subtracted to obtain the net effect size (ES), which was used together with the pooled SD of change to calculate the variance (ES = [mean BA − mean PL]/SD); to interpret the magnitude of the ES, Cohen’s criteria were followed: <0.2, trivial; 0.2–0.5, small; 0.5–0.8, moderate; and >0.8, large [40].

The I^2^ statistic was calculated as an indicator of the percentage of observed total variation within studies due to real heterogeneity rather than chance. I^2^ values are included from 0 to 100%, representing a small amount of inconsistency between 25% and 50%, a medium amount of heterogeneity between 50% and 75%, and a large amount of heterogeneity when the I^2^ value was higher than 75%. In this sense, low, moderate, and high adjectives would be accepted referring to I^2^ values of 25%, 50%, and 75%, respectively, although a restrictive categorization would not be adequate in all circumstances [41].

## 3. Results

### 3.1. Main Search

**Figure 4:**
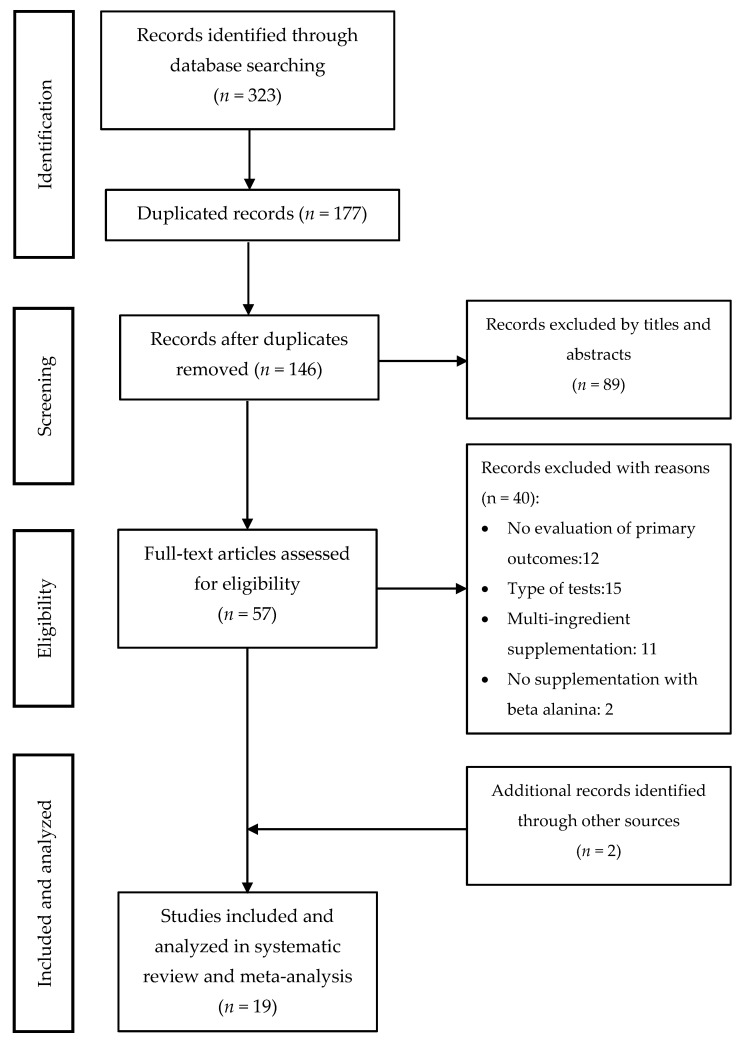
Studies included in the systematic review and meta-analysis.

The literature search through electronic databases identified 323 articles of which 177 were duplicates. The remaining 146 articles were filtered by title and abstract, and 57 studies remained to be read and analyzed. After a review of those 57 studies, 40 were eliminated because they did not meet the inclusion criteria. In the search for articles oriented by bibliographic references, two extra studies were added. As a result, 19 articles were included for the systematic review and meta-analysis. The search strategy and study selection are shown in Figure 4. Out of 19 studies, eight considered time in a TTT to assess the effect of BA supplementation on physical performance [29,42,43,44,45,46,47,48], nine used time (LTT) on a TTE test to assess the same effect [5,21,22,35,49,50,51,52,53], one considered the distance (LDT) on a TTE test [28], while one considered both the time (LTT) and the distance (LDT) on a TTE test [17] (Table 1).

### 3.2. Effect of BA on Time Trial Tests

**Figure 5:**
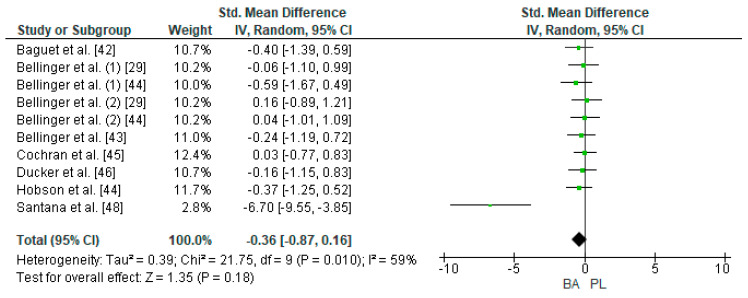
Forest plot comparing the effects of BA supplementation on Time Trial Tests. BA: beta-alanine; PL: placebo.

Eight studies were considered for this analysis [29,42,43,44,45,46,47,48]. However, two of them included two TTTs in the research design [29,44]. For the meta-analysis, the study by Bellinger et al. [29] was considered as two independent designs (TTT of 4 and 10 km on a cycle ergometer, respectively). In the same way, the study by Bellinger et al. [44] was considered as two independent designs (TTT of 4 and 10 km on a cycle ergometer, respectively). Thus, 10 studies were included in the meta-analysis that calculated the effect of BA supplementation on time in TTT. Figure 5 shows that BA supplementation generates a small and non-significant effect on physical performance in TTT (SMD, −0.36; 95% CI −0.87–0.16; *p* = 0.18). The meta-analysis showed moderate heterogeneity among the studies reviewed (I^2^ = 59%; *p* = 0.01). Out of the 10 studies analyzed, seven of them declared a beneficial effect of supplementation with BA on physical performance in TTT [29,42,43,46,47,48]. Out of these studies, the research of Santana et al. [48] presented a large ES (−6.70). On the other hand, three of the 10 studies showed a neutral or prejudicial effect after BA supplementation [44,45].

### 3.3. Effect of BA on the Limited Time Test

**Figure 6:**
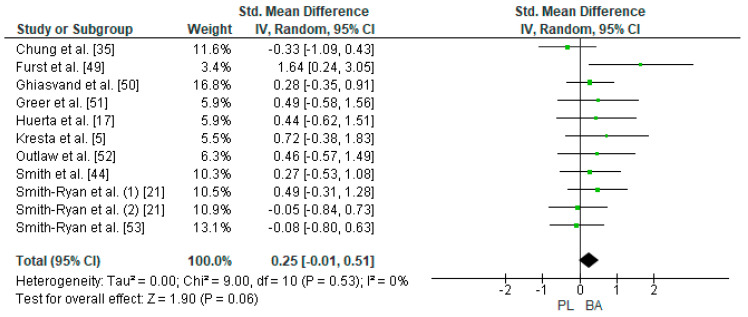
Forest plot comparing the effect of BA on Limited Time Test. BA: beta-alanine; PL: placebo.

Ten studies were considered for this analysis [5,17,21,22,35,49,50,51,52,53]. However, one of them included two experimental groups for the LTT in their research design [21]. For the meta-analysis, two experimental groups presented by Smith-Ryan et al. [21] were considered as two independent studies (LTT at 90% of VO_2_max on a treadmill for women and LTT at 90% of VO_2_max on a treadmill for men). This way, 11 studies were included in the meta-analysis that calculated the effect of BA supplementation on the TTE test. Figure 6 shows that BA supplementation generated a small and non-significant effect for time on the TTE test (SMD, 0.25; 95% CI −0.01–0.51; *p* = 0.06). A meta-analysis showed low heterogeneity among the reviewed studies (I^2^ = 0%; *p* = 0.53). Out of the 11 studied and analyzed, eight showed a positive effect of BA on time in the LTT [5,17,21,22,49,50,51,52]. Out of these studies, Furst et al. [49] showed a large ES (1.64). On the other hand, three of the 11 studies showed an unbeneficial effect after BA supplementation [21,35,53].

### 3.4. Effect of BA on the Limited Distance Test

**Figure 7:**
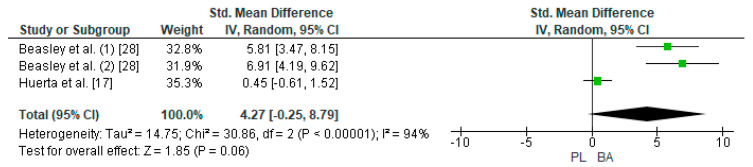
Forest plot comparing the effect of BA on Limited Distance Test. BA: beta-alanine; PL: placebo.

Two studies were considered for this analysis [17,28]. However, the study of Baesley et al. [28] included two experimental groups for the LDT in their research design (30 min on a rowing ergometer with 2.4 g/day of BA supplementation every 24 h and 30 min on a rowing ergometer with 4.8 g/day of BA supplementation every 48 h). In this way, three studies were included in the meta-analysis that calculated the effect of BA supplementation on the TTE test. Figure 7 shows that BA supplementation generates a large and non-significant effect on distance in the TTE test (SMD, 4.27; 95% CI −0.25–8.79; *p* = 0.06). The meta-analysis showed high heterogeneity among the studies reviewed (I^2^ = 94%; *p* = 0.00001). All studies analyzed declared a beneficial effect of supplementation with BA on physical performance in LDT [17,28].

### 3.5. Effect of BA Supplementation on Secondary Outcomes

Of the total of 19 studies included in the systematic review and meta-analysis, 17 of them reported on different parameters of physical performance. These parameters were defined as secondary outcomes and included blood lactate concentration ([La]), VO_2_max, RPE, and HR [32].

The meta-analysis of [La] (mmol·L^−1^) included 11 studies [5,17,21,28,29,35,43,46,47,48,49]. The total number of cases supplemented with BA included 128 participants, while 121 participants were supplemented with PL. The meta-analysis showed that BA supplementation generated a trivial and non-significant effect on [La] post effort (SMD, 0.16; 95% CI −0.35–0.67; *p* = 0.53), while moderate heterogeneity was present among the reviewed studies (I^2^ = 71%; *p* = 0.0001). A meta-analysis of absolute VO_2_max (LO_2_·min^−1^) included nine studies [5,22,28,43,45,50,51,52,53]. The total number of cases supplemented with BA included 109 participants, while the PL group comprised 104 participants. The meta-analysis showed that BA supplementation generated a trivial and non-significant effect on absolute VO_2_max (SMD, 0.17; 95% CI −0.11–0.45; *p* = 0.24), and low heterogeneity was observed among the studies (I^2^ = 6%; *p* = 0.39). The meta-analysis of RPE [36] included four studies [17,28,35,43]: the total number of cases supplemented with BA included 48 participants, while those supplemented with PL comprised 46. The meta-analysis showed that BA supplementation generated a trivial and non-significant effect on RPE (SMD, 0.03; 95% CI −0.52–0.58; *p* = 0.92), and low heterogeneity was observed in the studies (I^2^ = 42%; *p* = 0.14). Finally, the meta-analysis for HR included three studies [17,28,35], and the total of number of cases supplemented with BA included 39 participants, while those supplemented with PL comprised 38. The meta-analysis showed that BA supplementation generates a small and non-significant effect on HR (SMD, 0.30; 95% CI −0.66 to −1.26; *p* = 0.54), and a high heterogeneity was observed among the studies reviewed (I^2^ = 75%; *p* = 0.008).

### 3.6. Paresthesia

At the end of this review, out of the 19 studies included in the systematic review and meta-analysis, four of them reported paresthesia [21,22,29,51] (Table 1).

## 4. Discussion

In connection with the studies included in the systematic review and meta-analysis, the results showed that BA supplementation presents an ES ranging from a small (0.2–0.5) to a large magnitude (>0.8) in aerobic–anaerobic transition zones. At the same time, the results showed that changes in physical performance are associated with both acute and chronic BA supplementation, while the administered doses ranged from 1.5−6.4 g/day in periods ranging from 1 h before physical tests (acute supplementation) to 10 weeks with one or several doses during the day (chronic supplementation).

At the end of this review, several studies concluded that the increase in physical performance after BA supplementation is due to an increase in muscular CA concentrations [21,42,53]. The ergogenic effect that generates increased CA is associated with intracellular regulation of pH (buffer), an increase in Calcium (Ca^2+^) sensitivity in type I and II muscle fibers, and an increase in Ca^2+^/H^+^ ion exchange; as a consequence, these events showed an increase in muscular contractility [1]. For this reason, direct supplementation with CA has been studied with inconclusive results [14,54,55], since CA is degraded into BA and L-histidine in the stomach [5]. Specifically, the low effectiveness of direct supplementation with CA is related to the fact that L-histidine has a larger presence in plasma than BA [1]. Because of this, BA supplementation shows better results than CA supplementation [44,50].

At the end of this review, the only secondary effect reported and associated with BA supplementation was paresthesia [21,22]. This is a sensation of flushing associated with an irritant tingling in the ears, scalp, hands, and torso [23]. The process responsible for paresthesia is the release of L-histidine to form CA [9,12,27]. Paresthesia is transitory and can be avoided by dosing and ingesting BA in smaller portions throughout the day [9,12,27].

### 4.1. Effect of BA on the Time Trial Test and Time to Exhaustion Test

BA supplementation and the subsequent increase in CA could diminish H^+^ circulation and prevent the drop in intracellular pH during high-intensity exercise [50]. In fact, CA has been described as the main buffering substance of H^+^ at the muscular level [56]. Previous studies have stated that blood and muscular acidosis limit muscular contractility, which would favor the onset of fatigue [17,29,47,50]. At the same time, due to an increase in Ca^2+^ sensitivity to type I fibers, it has been mentioned that BA supplementation can improve muscular contractile properties, delaying fatigue onset [17,57].

As mentioned above, the performance increase in aerobic–anaerobic transition zones is associated with greater availability of muscular CA [20,50]. This way, evidence has shown that prolonged BA supplementation in doses ranging from 2.0–6.4 g/day for 4–10 weeks can increase CA concentrations between 64–80% [9]. In connection with acute supplementation in aerobic–anaerobic transition zones, evidence is scarce [17]. In this regard, Huerta et al. [17] performed supplementation with 30 mg·kg^−1^ body mass (1.5–2.1 g/day) of BA 60 min prior to a TTE test. These researchers obtained an average increase of 40.5 s at the end of the study (*p* < 0.05). Despite that, and due to the limited evidence relating acute supplementation with BA on physical performance in aerobic–anaerobic transition zones, it is impossible to guarantee a real effect in this physiological zone. However, the increase in physical performance observed in this review is supported by greater bioavailability of CA, an increase that is observed shortly after the intake of BA [58]. This raises the possibility of studying the acute effects of BA using different protocols and observing the real effects in aerobic–anaerobic transition zones.

The ES for distance on the TTE test was large (ES = 4.27), while TTT and time on the TTE test was small (ES = −0.36 and 0.25, respectively). In light of these results, these last values show a small effect of BA supplementation on physical performance in aerobic–anaerobic transition zones. However, considering that an elite athlete’s performance is bound by extremely tight margins (probably difficult to measure statistically), in real practice, a small ES could be of great importance, since it has been proven that in world finals, differences lower than 3% can be found between first and last place [1].

### 4.2. Effect of BA on Secondary Outcomes

BA supplementation could prevent the drop in intracellular pH during high-intensity exercise (due to an increase in muscular CA bioavailability) and, as a consequence, generate less lactate accumulation with the same intensity of physical exercise [48,50]. Regarding lactate accumulation, it is important to mention that this is not the cause of H^+^ accumulation, but a high intensity of exercise produces a decrease in pH and an increase in intramuscular and blood [La] simultaneously, transforming lactate in a good marker of physical effort [8]. Despite the theoretical background, the meta-analysis showed a trivial effect on [La] post effort (ES = 0.16).

The influence of BA supplementation on aerobic performance has been widely studied [14,20,27,59]; however, the meta-analysis showed a trivial effect of BA on VO_2_ (ES = 0.17) [51,60]. Apparently, the increase in VO_2_ is less dependent on the buffer qualities that BA supplementation produces [20]. It is possible that the improvement in VO_2_ reported in some studies included in the meta-analysis is more connected to physical training in aerobic–anaerobic transition zones than to BA supplementation [61,62].

In connection to RPE, some studies have shown a good correlation between RPE and HR during physical exercise in healthy subjects (1 point of RPE equals approximately 10 bpm). More so, the metabolic thresholds have been associated with specific values on the Borg scale [36]. Likewise, it has been shown that a lower value of RPE for the same workload entails a metabolic adaptation after the training process [63]. Despite these lines of theoretical evidence, the studies included in the meta-analysis showed a trivial effect on RPE reported by the participants (ES = 0.03). This value can be derived from the level of demand experienced by the participant; it is also possible that they exerted themselves to the maximum effort in all tests, reaching the upper limits of the RPE scales used [36].

As a consequence improved cardiac contractility, it has been described that CA can increase HR [53]. In addition, intracellular pH has proven to be a modulator of cardiac function, increasing the entrance of Ca^2+^ during action potentials, facilitating cardiac contraction [64]. This information makes it possible to anticipate an increase in HR after BA supplementation [53]. However, HR is dependent on the intensity of physical effort; hence, if the participants exerted themselves to the maximum in all tests, it is likely that post-effort HR values would not show major variations when supplementing with BA (ES = 0.30).

Finally, due to a limited number of studies, only the secondary outcomes mentioned above were used. Subdividing the 10 TTT studies and 11 TTL studies to perform a meta-analysis by gender, age, exercise modalities, or physical activity level would have generated a bias in the information obtained [38].

### 4.3. Limitations

The main limitations of this research were the access to information and unspecific data reported by some studies included in the systematic review and meta-analysis. However, the limitations were solved by contacting the authors of each study. Only one document was not included because no answer was received. Another important limitation in this review was the limited number of studies that used TDL as a primary outcome [17,28].

## 5. Conclusions

Both acute and chronic supplementation with BA in doses of 1.5–6.4 g/day showed a small and non-significant effect on physical performance in aerobic–anaerobic transition zones. Physiologically, this positive change is due to the buffer effects generated by the larger bioavailability of intracellular CA, which allows for a delay in the onset of fatigue in the TTT and TTE tests within this specific physiologic zone. That is why small changes in individual performance must be considered, since they can be the difference between success and failure among high-level and elite athletes.

Furthermore, the findings showed evidence that acute supplementation with BA is scarce, generating alternatives for researchers to study the effect of this form of supplementation with different BA doses and formats on performance in aerobic–anaerobic transition zones.

## 6. Practical Applications

Coaches and athletes looking for an ergogenic aid to enhance physical performance in aerobic–anaerobic transition zones should consider both acute and chronic supplementation with BA. The dosage can range from 30 mg·kg^−1^ of body mass in acute supplementation to 6.4 g/day in chronic supplementation. The latter may be administered in several doses per day. However, it is advisable to check the dosage and supplementation formats with qualified professionals.

Finally, in order to avoid the presence of paresthesia after supplementation with BA, it is recommended that BA be dosed and ingested in small portions throughout the day (the amount suggested for these doses is 1.6 g of BA per dose) [9]. The second recommendation to avoid paresthesia is to also ingest a large amount of carbohydrates 60 min before ingesting BA (the suggested carbohydrate load is 2 g·kg^−1^ of body mass) [17].

## Figures and Tables

**Table 1:** Characteristics of the studies that connect BA supplementation with physical performance in aerobic–anaerobic transition zone.

Author	Objective	Subjects	Variables	Test	Supplementation Protocol	Results	Performance in PO
Chronic effect of BA supplementation in aerobic–anaerobic transition zone							
Baguet et al. [42]	To investigate if performance is related to the muscle CA content and if BA suppl improves performance in highly trained rowers.	C/A: Rowers M = 16; F = 1 (EG = 8; CG = 9) A: 22.9 ± 4.2 years	I: EG: BA + training CG: PL + training D: PO: TTT	TTT: 2000 m rowing ergometer	Oral suppl of 7 w Total dose 7 w: 245 g EG: BA 5 g/day (5 doses of 1 g, c/2 h) CG: maltodextrin	Time (s) post test: EG = 386.5 vs. CG = 391.5; *p* > 0.05 The authors declared paresthesia: no	↔
Beasley et al. [28]	To investigate the effect of two BA dosing strategies on 30 min rowing and subsequent sprint performance.	RA: Rowers M = 27 (EG_1_ = 9; EG_2_ = 9; CG = 9) A: 24.0 ± 5.0 years	I: EG: BA + training CG: PL + training D: PO: LDT SO: VO_2_max, [La], RPE, HR	LDT: 30 min rowing ergometer	Oral suppl of 28 d Total dose 28 d: 67.2 g EG_1_: BA 2.4 g/day (1 dose of 2.4 g, e/24 h) EG_2_: BA 4.8 g/day (1 dose of 4.8 g, e/48 h) CG: corn flour	Distance (m) post test: GE_1_ = 7579 vs. CG = 7228; *p* > 0.05 GE_2_ = 7575 vs. CG = 7228; *p* > 0.05 VO_2_max (LO_2_·min^−1^) post test: GE_1_ = 3.63 vs. CG = 3.33; *p* > 0.05 GE_2_ = 3.50 vs. CG = 3.33; *p* > 0.05 [La] (mmol·L^−1^) post test: GE_1_ = 10.0 vs. CG = 9.1; *p* > 0.05 GE_2_ = 8.8 vs. CG = 9.1; *p* > 0.05 RPE (1–10) post test: GE_1_ = 9.7 vs. CG = 9.6; *p* > 0.05 GE_2_ = 9.3 vs. CG = 9.6; *p* > 0.05 HR (bpm) post test: GE_1_ = 190 vs. CG = 185; *p* > 0.05 GE_2_ = 182 vs. CG = 185; *p* > 0.05 The authors declared paresthesia: no	↔
Bellinger et al. [43]	To investigate the effects of BA suppl on the resultant blood acidosis, lactate accumulation, and energy provision during supramaximal-intensity cycling, as well as the aerobic and anaerobic contribution to power output during a 4000 m cycling time trial.	C/A: Cyclists M = 17 (EG = 9; CG = 8) A: 24.5 ± 6.2 years	I: EG: BA + training CG: PL + training D: PO: TTT SO: VO_2_max, [La], RPE	TTT 4000 m cycle ergometer	Oral suppl of 28 d Total dose of 28 d: 179.2 g EG: BA 6.4 g/day (4 dose of 1.6 g, in every meal) CG: dextrose monohydrate	Time (s) post test: EG = 355.6 vs. CG = 360.4; *p* < 0.05 VO_2_max (LO_2_·min^−1^) post test: EG = 4.45 vs. CG = 4.44; *p* > 0.05 [La] (mmol·L^−1^) post test: EG = 15.1 vs. CG = 15.2; *p* > 0.05 RPE (6–20) post test: EG = 18.8 vs. CG = 18.8; *p* > 0.05 The authors declared paresthesia: no	↑
Bellinger et al. [29]	To assess the efficacy of BA suppl on cycling time trial of different length in the same group of trained cyclists and to contrast the effects of BA supply on a supramaximal time to fatigue test.	C/A: Cyclists M = 14 (EG = 7; CG = 7 A: 24.8 ± 6.7 years	I: EG: BA + training CG: PL + training D: PO: TTT SO: [La]	TTT 4 km and 10 km cycle ergometer	Oral suppl of 28 d Total dose 28 d: 179.2 g EG: BA 6.4 g/day (4 dose of 1.6 g, with every meal) CG: dextrose monohydrate	4 km Time (s) post test: EG = 356.6 vs. CG = 357.8; *p* > 0.05 [La] (mmol·L^−1^) post test: EG = 15.3 vs. CG = 15.4; *p* > 0.05 10 km Time (s) post test: EG = 938.1 vs. CG = 929.9: *p* > 0.05 [La] (mmol·L^−1^) post test: EG = 11.0 vs. CG = 12.9; *p* > 0.05 The authors declared paresthesia: yes	4 km ↔ 10 km ↔
Bellinger et al. [44]	To investigate the effects of BA suppl only, and in combination with sprint-interval training, on training intensity, and energy provision and performance during exhaustive supramaximal-intensity cycling and a 4 and 10 km time trial.	C/A: Cyclists M = 14 (EG = 7; CG = 7) A: 25.4 ± 7.2 years	I: EG: BA + training CG: PL + training D: PO: TTT	TTT 4 km and 10 km cycle ergometer	Oral suppl of 9 w Total dose 9 w: 221.2 g EG: BA 6.4 g/day for 4 w (4 doses of 1.6 g, w/every meal) + BA 1.2 g/day for 5 w (3 doses of 400 mg, every 3 to 4 h) CG: dextrose monohydrate	4 km Time (s) post test: EG = 339.7 vs. CG = 350.1; *p* > 0.05 10 km Time (s) post test: EG = 918.5 vs. CG = 916.6; *p* > 0.05 The authors declared paresthesia: no	4 km ↔ 10 km ↔
Chung et al. [35]	To investigate whether BA suppl can increase muscle CA stores in endurance-trained athletes, and whether CA loading can improve their endurance performance.	C/A: Cyclists and triathletes M = 27 (EG = 14; CG = 13) A: 30.9 ± 7.9 years	I: EG: BA + training CG: PL + training D: PO: LTT SO: [La], RPE, HR	LTT incremental cycle ergometer (from 50 W, ≥60 rpm until exhaustion)	Oral suppl of 6 w Total dose 6 w 268.8 g EG: BA 6.4 g/day (4 doses of 1.6 g, with every meal) CG: maltodextrin	Time (s) post test: EG = 3696 vs. CG = 3780; *p* > 0.05 [La] (mmol·L^−1^) post test: EG = 9.7 vs. CG = 7.3; *p* > 0.05 RPE (6–20) post test: EG = 19.0 vs. CG = 18.8; *p* > 0.05 HR (bpm) post test: EG = 181 vs. CG = 180; *p* > 0.05 The authors declared paresthesia: no	↔
Cochran et al. [45]	To increase skeletal-muscle CA and augment muscle buffering capacity during a 6 week sprint interval training intervention.	PA: Healthy subjects M = 24 (EG = 12; CG = 12) A: 22.5 ± 2.0 years	I: EG: BA + training CG: PL + training D: PO: TTT SO: VO_2_max	TTT 250 KJ cycle ergometer	Oral suppl of 10 w Total dose 10 w: 224 g EG: BA 3.2 g/day (2 doses of 1.6 g, every 12 h) CG: dextrose	Time (s) post test: EG = 1130 vs. CG = 1125; *p* > 0.05 VO_2_max (mL O_2_·kg^−1^·min^−1^) post test: EG = 52.2 vs. CG = 55.4; *p* > 0.05 The authors declared paresthesia: no	↔
Ducker et al. [46]	To assess if beta-alanine suppl could improve 2000 m rowing-ergometer performance in well-trained male rowers.	C/A: Rowers M = 16 (EG = 7; CG = 9) A: 26.0 ± 9.0 years	I: EG: BA + training CG: PL + training D: PO: TTT SO: [La]	TTT 2000 m rowing ergometer	Oral suppl of 28 d Total dose 28 d: ~ 168 a 196 g (80 mg·kg^−1^·d^−1^) EG: BA ~ 6–7 g/day (4 doses of 1.5–1.75 g, with every meal) CG: glucose	Time (s) post test: EG = 391.0 vs. CG = 393.4; *p* > 0.05 [La] (mmol·L^−1^) post test: EG = 12.5 vs. CG = 12.4; *p* > 0.05 The authors declared paresthesia: no	↔
Furst et al. [49]	To investigate the effect of BA suppl on exercise endurance and executive function in a middle-aged human population.	N/T: Healthy subjects M = 8; F = 4 (EG =7; CG = 5) A: 60.5 ± 8.6 years	I: EG: BA + training CG: PL + training D: PO: LTT SO: [La]	LTT at 70% VO_2_peak cycle ergometer	Oral suppl of 28 d Total dose 28 d: 67.2 g EG: BA 2.4 g/day (3 doses of 800 mg, with every meal) CG: microcrystalline cellulose	Time (s) post test: EG = 876 vs. CG = 522; *p* > 0.05 [La] (mmol·L^−1^) post test: EG = 6.6 vs. CG = 4.2; *p* > 0.05 The authors declared paresthesia: no	↔
Ghiasvand et al. [50]	To assess the effects of alanine suppl on VO_2max_, time to exhaustion, and lactate concentrations in physical education male students.	RA: Healthy college students M = 39 (EG = 20; CG = 19) A: 21.5 ± 1.1 years	I: EG: BA + training CG: PL + training D: PO: LTT SO: VO_2_max, [La]	LTT incremental cycle ergometer (from 30 W, ≥70 rpm to exhaustion)	Oral suppl of 6 w Total dose 6 w: 84 g EG: BA 2 g/day (5 doses of 400 mg, with every meal) CG: dextrose	Time (s) post test: EG = 992.4 vs. CG = 926.5; *p*: < 0.05 VO_2_max (L O_2_·min^−1^) post test: EG = 2.79 vs. CG = 2.81; *p* < 0.05 [La] (mg·dL^−1^) post test: EG = 27.9 vs. CG = 36.0; *p* < 0.05 The authors declared paresthesia: no	↑
Greer et al. [51]	To determine the effect of 30 days of BA suppl on peak aerobic power and ventilatory threshold in aerobically fit males.	C/A: Aerobically fit males M = 14 (EG = 7; CG = 7) A: 28.8 ± 9.8 years	I: EG: BA + training CG: PL + training D: PO: LTT SO: VO_2_max	LTT cycle ergometer	Oral suppl of 30 d Total dose 30 d: 159 g EG: BA 3 g/day for 7 d (2 doses of 1.5 g, every 12 h) + BA 6 g/day for 23 d (4 doses of 1.5 g, with every meal) CG: maltodextrin	Time (s) post test: EG = 1304 vs. CG = 1125; *p* > 0.05 VO_2_max (L O_2_·min^−1^) post test: EG = 4.14 vs. CG = 3.97; *p* > 0.05 The authors declared paresthesia: yes	↔
Hobson et al. [47]	To examine the effect of BA only and BA with sodium bicarbonate suppl on 2000 m rowing performance.	C/A: Rowers M = 20 (EG = 10; CG = 10) A: 23.0 ± 4.0 years	I: EG: BA + training CG: PL + training D: PO: TTT SO: [La]	TTT 2000 m rowing ergometer	Oral suppl of 30 d Total dose 30 d: 192 g EG: BA 6.4 g/day (4 doses of 1.6 g, every 3–4 h) CG: maltodextrin	Time (s) post test: EG = 410.3 vs. CG = 416.4; BA probability on PL: 96% effect (+) [La] (mmol·L^−1^) post test: EG = 14.7 vs. CG = 14.5; *p* > 0.05 The authors declared paresthesia: no	↑
Kresta et al. [5]	To examine the short-term and chronic effects of BA suppl with and without creatine monohydrate on body composition, aerobic, and anaerobic exercise performance and muscle CA and creatine levels in college-aged recreationally active females.	FA: Healthy college students F = 15 (EG = 8; CG = 7) A: 21.5 ± 2.8 years	I: EG: BA + training CG: PL + training D: PO: LTT SO: VO_2_max	LTT incremental cycle ergometer (from 50 W, ≥70 rpm to exhaustion)	Oral suppl of 28 d Total dose 28 d: ~ 170.8 g (0.1 g·kg^−1^·d^−1^) EG: BA ~ 6.1 g/day (around 4 doses of 800 mg, every 4 h) CG: dextrose and maltodextrin	Time (s) post test: EG = 1293 vs. CG = 1083; *p* > 0.05 VO_2_max (mL O_2_·kg^−1^·min^−1^) post test: EG = 41.53 vs. CG = 37.90; *p* > 0.05 The authors declared paresthesia: no	↔
Outlaw et al. [52]	To evaluate the cumulative effect of resistance training and BA suppl on aerobic and anaerobic performance markers, as well as body composition, in collegiate females.	S/E: Untrained collegiate females F = 15 (EG = 7; CG = 8) A: 21.0 ± 2.2 years	I: EG: BA + training CG: PL + training D: PO: LTT SO: VO_2_max	LTT treadmill	Oral suppl of 8 w Total dose 8 w: 108.8 g (32 doses) EG: BA 3.4 g/day (1 single dose before training) CG: maltodextrin	Time (s) post test: EG = 629.1 vs. CG = 591.1; *p* > 0.05 VO_2_max (mL O_2_·kg^−1^·min^−1^) post test: EG = 41.2 vs. CG = 38.6; *p* > 0.05 The authors declared paresthesia: no	↔
Santana et al. [48]	To investigate the effects of BA suppl on a 10 km running time trial and lactate concentration in physically active adults.	PA: Healthy subjects M = 16 (EG = 8; CG = 8) A: 29.4 ± 3.9 years	I: EG: BA + training CG: PL + training D: PO: TTT SO: [La]	TTT 10 km treadmill	Oral suppl of 23 d Total dose 23 d: 115 g EG: BA 5 g/day (around 3 doses of 1.6 g, every 3 h) CG: resistant starch	Time (s) post test: EG = 3210 vs. CG = 3480; *p* < 0.05 [La] (mmol·L^−1^) post test: EG = 6.8 vs. CG = 10.8; *p* < 0.05 The authors declared paresthesia: no	↑
Smith et al. [22]	To evaluate the effects of 28 days of BA suppl on markers of oxidative stress.	RA: Healthy women F = 24 (EG = 13; CG = 11) A: 21.7 ± 2.1 years	I: EG: BA + training CG: PL + training D: PO: LTT SO: VO_2_max	LTT incremental in treadmill (from 10 km∙h^−1^ to exhaustion)	Oral suppl of 28 d Total dose 28 d: 134.4 g EG: BA 4.8 g/day (3 doses of 1.6 g, in intervals) CG: maltodextrin	Time (s) post test: EG = 405 vs. CG = 388; *p* > 0.05 VO_2_max (L O_2_·min^−1^) post test: EG = 2.71 vs. CG = 2.64; *p* > 0.05 The authors declared paresthesia: yes	↔
Smith-Ryan et al. [21]	To evaluate the effects of BA suppl on high-intensity running performance and critical velocity anaerobic running capacity.	RA: Healthy subjects M and F = 50 (EG = 26; CG = 24) A: 21.9 ± 2.7 years	I: EG: BA + training CG: PL + training D: PO: LTT SO: [La]	LTT at 90% V_max_ treadmill	Oral suppl of 28 d Total dose 28 d: 134.4 g EG: BA 4.8 g/day (3 doses of 1.6 g, in intervals) CG: maltodextrin	Women Time (s) post test: EG = 313.8 vs. CG = 240.5; *p* > 0.05 [La] (mmol·L^−1^) post test: EG = 13.15 vs. CG = 13.8; *p* > 0.05 Men Time (s) post test: EG = 317.0 vs. CG = 322.6; *p* > 0.05 [La] (mmol·L^−1^) post test: EG = 15.7 vs. CG = 13.8; *p* > 0.05 The authors declared paresthesia: yes	↔
Smith-Ryan et al. [53]	To determine the effect of 28 days of BA suppl on work physical capacity test in heart rate threshold.	FA: Healthy subjects M and F = 30 (EG = 15; CG = 15) A: 21.0 ± 2.1 years	I: EG: BA + training CG: PL + training D: PO: LTT SO: VO_2_max	LTT incremental cycle ergometer (from 20 W, ≥60 rpm until exhaustion)	Oral suppl of 28 d Total dose 28 d: 1792 g EG: BA 6.4 g/day (4 dose of 1.6 g, every 3–4 h) CG: maltodextrin	Time (s) post test: EG = 690.5 vs. CG = 703.6; *p* > 0.05 VO_2_max (mL O_2_·kg^−1^·min^−1^) post test: EG = 39.1 vs. CG = 43.4; *p* > 0.05 The authors declared paresthesia: no	↔
Acute effect of BA supplementation on aerobic–anaerobic transition zone							
Huerta et al. [17]	To determine the acute effect of BA suppl on a limited time test at maximum aerobic speed on endurance athletes.	C/A: High-level athletes M and F = 7 (EG = 7; CG = 7) A: 24.2 ± 4.4 years	I: EG: BA + training CG: PL + training D: PO: TTE (LDT and LTT) SO: [La], RPE, HR	TTE (LTT and LDT) at maximum aerobic speed in athletic track	Oral suppl Total dose: 30 mg·kg^−1^ body mass EG: BA from 1.5–2.1 g/day 60 min before TTE (LDT and LTT) CG: simple carbohydrates	Time (s): EG = 366.5 vs. CG = 326.0; *p* < 0.05 Distance (m): EG = 1828.6 vs. CG = 1651.4; *p* > 0.05 [La] (mmol·L^−1^): EG = 14.80 vs. CG = 13.84; *p* > 0.05 RPE (1–10): EG = 8.28 vs. CG = 7.60; *p* > 0.05 HR (bpm): EG = 185.4 vs. CG = 178.8; *p* > 0.05 The authors declared paresthesia: no	Time ↑ Distance ↔

A: age; BA: beta-alanine; bpm: beat per minute; C/A: competitive athlete; CA: carnosine; CG: control group; d: days; D: dependent variable; e/: every; EG: experimental group; EG_1_: experimental group 1; EG_2_: experimental group 2; g/day: grams per day; g: grams; h: hours; HR: heart rate; I: independent variable; kg: kilograms; KJ: Kilo Joule; km: kilometers; L: liters; LDT: limited distance test; LTT: limited time test; M: male; F: female; m: meters; mg: milligrams; mg·dL^−1^: milligrams per deciliter; mg·kg^−1^: milligrams per kilogram; min: minutes; mmol·L^−1^: millimole per liter; N/T: no training; PA: physically active; PL: placebo; PO: primary outcome; RA: recreational athlete; RPE; ratings of perceived exertion; s: seconds; SO: secondary outcome; suppl: supplementation; T: time; TTE: time to exhaustion; TTT: time trial test; Vmax: maximum velocity; VO_2_: oxygen uptake; VO_2_max: maximal oxygen uptake; vs: versus; VT: ventilatory threshold; W: watt; w: weeks; [La]: blood lactate concentration; *p* < 0.05: significant change; *p* > 0.05: non-significant change; ~: approximate; ↑: positive effect; ↔: no effect.
